# A patient-centered evaluation of a novel medical student-based patient navigation program

**DOI:** 10.1016/j.pec.2023.108131

**Published:** 2023-12-29

**Authors:** Jeremy Wilson, Derrick Lau, Eva Kristoferson, Ellen Ginzler, Naureen Kabani

**Affiliations:** SUNY Downstate Health Sciences University, Brooklyn, NY, USA

**Keywords:** Patient navigation, Medical Student, Lupus, Rheumatology, Health Disparities, Health equity, Patient-centered research, Care coordination

## Abstract

**Objectives:**

Understand the patient experience of a pilot medical student-based patient navigator (PN) program. (2) Assess areas of improvement for further development as a model for expansion.

**Methods:**

This was a cross-sectional study assessing patients’ subjective experience of medical student navigators for rheumatological conditions. Current student navigators contacted enrolled patients by phone with both structured and free-response questions.

**Results:**

44 of 71 patients completed the questionnaire. 84% reported a satisfaction of ≥ 4 on a 5 point Likert scale. > 80% of patients felt that the program helped them better care for their health, feel more understood by their medical team, and feel cared for by their healthcare team. Medical student navigators were able to assist with most patient requests.

**Conclusions:**

Patients enrolled in our medical student PN program expressed high levels of satisfaction and felt better able to access health resources with the help of a navigator.

**Practice implications:**

Employing medical students as PNs may serve as a mutually beneficial intervention providing early clinical exposure to students while furthering patient access to care. Other institutions may benefit from similarly structured interventions.

## 1. Introduction

The first patient navigator (PN) program was implemented in Harlem Hospital in 1990 and focused on breast cancer screening in Black patients [1]. Since then, PN programs have become increasingly popular, however there is considerable heterogeneity in models. These programs can go by a number of names including patient assistance programs, system navigators, patient advocates, case coordinators, and health coaches. Each describes someone working to coordinate patient care and address barriers to healthcare [1,2]. Some specific examples include facilitating communication between patient and provider, appointment reminders, assisting with medication refills, providing educational resources, and providing emotional support [3]. These services, among others, have been shown to increase patient understanding of their disease and lead to improved patient outcomes [4–15]. The most robust data demonstrating the efficacy of PN programs comes from improving cancer screening and treatment [16], various primary care interventions [5,9], and HIV medication adherence [10].

Despite immense disparities in systemic lupus erythematosus (SLE) care for racial and ethnic minorities [17], there remains a paucity of PN programs serving those suffering from rheumatological diseases. Some studies, however, have shown efficacy of PN interventions in this population. For example, one study [14] implemented a rheumatology-specific PN program to address disease modifying anti-rheumatic drugs (DMARD) medication adherence. Another study [13] demonstrated that only PN, compared to peer-to-peer support and patient support groups, had a significant increase in measured self-efficacy and patient activation among Black patients with SLE. Similar studies also show increased self-efficacy in SLE when patients are paired with a PN [12]. Nonetheless, there are few rheumatology-specific patient navigation programs despite the known disparities in care and complex management issues in this population.

The role of PN can be filled by a number of people including lay people [18], case managers [19], social workers [20], nurses [21] and former patients with the same disease [22]. The vast majority of PNs are nurses or lay/community healthcare workers [16], however a few programs have successfully used medical students as PNs. One such program at Case Western Reserve University offered first year medical students the opportunity to serve as PNs as part of their Health Systems Science curriculum [23]. Focus groups of students from this cohort demonstrated remarkable impact on learners across multiple domains. Others have focused on the utilization of medical student navigators to teach empathy in medical school [2]. However, each of these approaches fail to assess the patient perception of these programs. Furthermore, there are very few studies that are centered on the patient’s experience of navigation outside of medical outcomes. One systematic review of PN programs for patients with chronic disease explicitly called for more studies that analyzed patients’ experiences [24]. One of the few studies that did examine patients’ navigator preferences demonstrated that patients valued not only the logistical role that their PNs could play, but the emotional support as well [25]. Further, this study highlighted the ways in which the PN was able to bridge the gap between the complex healthcare system and the biopsychosocial needs of the patient. However, as with much of the research on patient navigation, this study was conducted specifically in the setting of cancer care and utilized lay professionals as PNs.

Our novel PN program is built on the foundation and proven efficacy of prior models. This program was created in response to a 2021 grant from Aurinia Pharmaceuticals with a goal of “. increasing access to equitable healthcare for people living with lupus nephritis in underserved.

communities and create meaningful impact to patients [26].” Five sites were awarded $50,000 to implement a PN program at their institution. Four sites hired a part-time nurse or social worker to serve as PN. Ours was the only site to specifically hire medical students to act as PNs. Second-year medical students were asked to apply for the program, underwent training, and were provided a modest stipend for their time. In line with the goals of the grant, patients with known barriers to healthcare, history of missed appointments, and/or low health literacy were offered to be connected with a student navigator.

The novelty of our program lies in the fact that PNs were medical students who maintained relationships with their assigned patients over the course of two or more years. This confers a number of benefits for patients and students alike. For example, medical students are granted access to the electronic health record through their school which allows for easy onboarding. Additionally, hiring outside PNs requires over-coming tremendous bureaucratic hurdles from access to clinic sites to handling protected health information. This became a major obstacle faced by the other grant sites delaying their start by months. In contrast, since our medical students already operate within the hospital system, they were able to begin helping patients from day one. Further, students at this level of training have already begun generating a medical vocabulary that allows them to interpret medical records in a way that navigators lacking this training cannot. More so, students were eager to learn and forge relationships with patients as this served as an opportunity to experience early clinical encounters.

Given the complex nature of rheumatological care and urgent need for interventions addressing health disparities in this population, our study aimed to (1) assess the patient experience of using students as patient navigators and (2) identify areas for improvement of the program. This intervention is novel both in its use of medical students as navigators as well as the focused assessment of patient experience of navigation in a disease population in which PNs have been less frequently utilized. Based on our results, we also identified future areas of improvement and possible expansion into different clinics.

## 2. Methods

### 2.1. Participant recruitment

This study looked specifically at the first year cohort of patients enrolled in the rheumatology PN program. As this program was established in response to a grant to target underserved populations, patients were selected based on a history of recurrent missed appointments, inconsistent clinic follow up, or knowledge of significant barriers to care. Participant demographics are outlined in Table 1. Most patients in this clinic are well known to the medical team who were able to evaluate if they might benefit from a PN. The majority of the patients recruited had SLE and were Black. Patients were connected to their navigator in the clinic or occasionally over the phone following a visit. Navigators discussed with the patient the various ways in which they may be able to support the patient.

### 2.2. PN involvement

The first cohort of PNs included seven second year medical students of various backgrounds and languages spoken. Over the course of a few months, each PN was connected with between 8–10 patients each. In many cases, patients with English as their non-native language were able to be paired with navigators who spoke their native tongue. Students were able to access their patients’ medical records, attended rheumatology clinic bimonthly, and had support from clinic staff. PNs were given training regarding their role and were instructed to bring medical concerns to the attention of the fellows and attendings. PNs followed their patients for one year (sometimes more) often attending medical appointments, calling patients to check in, and helping connect patients with existing resources.

### 2.3. Data collection

We conducted a cross-sectional study of patients enrolled in the first cohort of the program. One to four months after completing their first year in the program, patients were contacted by phone to fill out a questionnaire (Table 2) to better understand their experience. During this time, many of the patients continued to benefit from the program, however, they were asked to reflect on the first year specifically. Patients were contacted by a member of the team who was not their assigned PN to limit response bias. The survey items were compiled based on common tasks PNs were able to assist patients with. There was also a section designed for open-ended patient responses. A preliminary survey was shared with select patients for review to incorporate patient feedback in the study design.

### 2.4. Data analysis

**Fig. 1.**
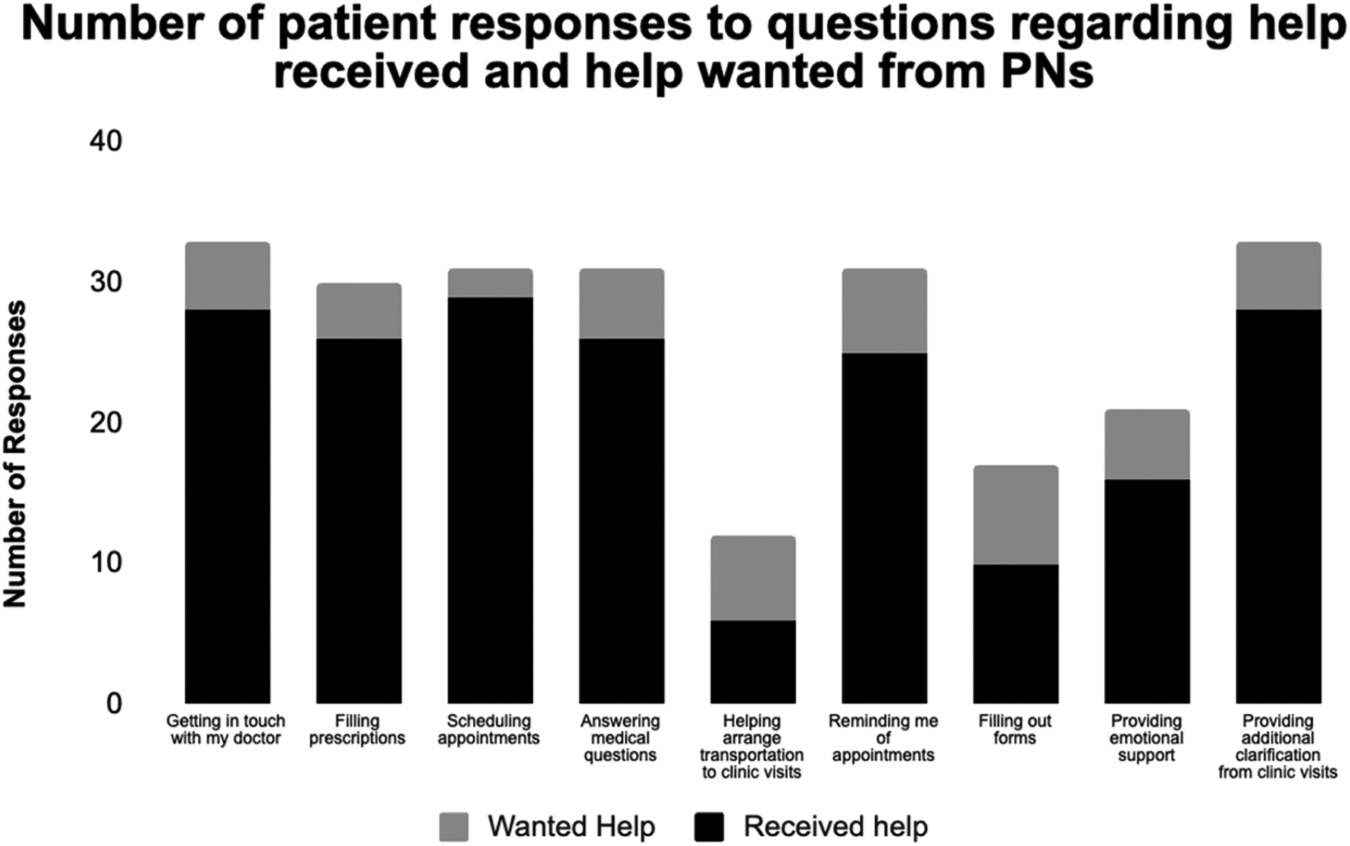
Numbers of patients receiving help with the specific tasks for which they requested assistance.

Survey results were downloaded from Google Forms and imported into Google Sheets. Responses were tabulated and percentage of responses corresponding to the desired value were calculated using formulas and functions in Google Sheets. Results were then imported into GraphPad Prism v9 to be formatted visually (Fig. 1).

For open-ended questions, data were analyzed using inductive thematic analysis as outlined by Braun and Clarke [27]. Two members of the research team (JW, DL) served as independent reviewers of the data. Reviewers began by familiarizing themselves with the survey responses. They then independently open coded responses which were subsequently grouped into initial themes. These themes were reviewed to ensure that they accurately depicted the codes and the data as a whole. Strong consensus between reviewers regarding themes was reached and representative theme titles were generated. Representative quotations for each theme were selected and agreed upon by members of the research team. One reviewer (DL) utilized Delve in vivo qualitative analysis software [28] to ensure accuracy of coding and that no key themes were missed. Data saturation was achieved with 39 of 44 patients providing free-response answers [29].

## 3. Results

### 3.1. Quantitative results

Out of the 71 patients contacted, 44 completed the questionnaire (62% response rate), with relatively equal participation between navigator assignments (ranging between 5–7 patients per navigator). When asked about their satisfaction with the program, 84% reported a satisfaction of ≥ 4 on a 5 point Likert scale (where 4 is satisfied and 5 is very satisfied).

Analysis of patient responses to which areas they wanted/received help showed that, for those interested in assistance, 94% of navigators were able to schedule appointments, 85% were able to get in touch with the doctor, 87% could assist with filling prescriptions, 85% were able to provide additional clarification from clinic visits, 84% were able to answer medical questions, 81% could remind patients of appointments, 76% were able to provide emotional support, and 59% were able to assist with filling out forms. The category with lowest percentage of assistance was arranging transportation to clinic visits where 50% felt that the navigators met their needs. These data reflect the percentage of patients who initially indicated interest in receiving help and ended up receiving that assistance. Patients who did not ask for help in a specific area, but nonetheless received that assistance were excluded from this analysis. These results are summarized in Fig. 1.

The responses for each patient are individually tabulated in Table A.1 (Appendix A) to show which patients requested and received help in specific areas.

When asked about the lasting impact our program had, 91% of patients agreed or strongly agreed they felt more cared for by their healthcare team, 84% agreed or strongly agreed they were more motivated to better care for their health as a result of the program, and 84% agreed or strongly agreed they felt their healthcare team now better understands the challenges they face in daily life (where agreed or strongly agreed was a 4 or 5 on a 5-point Likert scale, respectively).

### 3.2. Qualitative results

#### 3.2.1. Positive feedback

When asked about the most helpful aspect of having a PN, patients better elucidated some of the benefits of the program. The following themes emerged from our qualitative analysis of the free response data.

##### 3.2.1.1. Theme 1. Ease of contacting their doctor.

A number of patients specifically highlighted that they benefited tremendously from having a direct link to their doctor through their PN. Patients emphasized that they did not have their doctor’s phone number or email and that contacting them through the hospital phones takes too long. Having a person to advocate for them directly made getting treatment much easier. One patient emphasized this point, “There were times when I had pain and could not get in touch with my doctor, but I was always able to get in touch with [PN] and he helped advise me what to do.”

Many patients took note of how quickly they were able to have responses to questions or concerns they had. One patient described it as follows: “When I had an issue, she was able to get in touch with my doctor and find out the answer in such a quick way that I never would have been able to do on my own. For example, I’ve been having headaches and lately they have been affecting my eyes. I texted [PN] and she answered in 5 minutes and was giving me answers really quickly. She asked me medical questions before speaking with the doctor and resolved my issue within the hour. I was very impressed with her medical knowledge and responsiveness.”

##### 3.2.1.2. Theme 2. Arranging appointments and prescription refill assistance.

Other patients pointed to the aid they received with previously cumbersome tasks, such as filling prescriptions and setting up appointments, as a particularly strong benefit of the program. Patients noted that sometimes their prescription would run out and rather than having to make an appointment or call the office (which can have delays), their PN was able to contact their doctor to have the prescription filled in a timely manner. This often led to alleviation of pain or other symptoms much quicker than in the past. Furthermore, PNs in the program were given direct access to the office manager who could schedule appointments for patients in a timely manner. Instead of having to call the appointment call center and waiting for the next available appointment (which may not be for months), patients could contact their PN who had authority to help schedule their appointment even if the clinic was booked. As one patient put it, “When I need something she (my PN) was there and got stuff done in a timely manner. She was most helpful in filling prescriptions and setting up appointments for me.” Another said, “I needed help getting an appointment made and I reached out to her (my PN) and she took care of it. She helped me with something I couldn’t do myself. She has made things a lot easier for me which I really am thankful for.”

##### 3.2.1.3. Theme 3. Addressing life stressors.

Additionally, patients appreciated having someone on their medical team who could fill in some of the gaps that serve as barriers to their care. For example, two patients described how their PN was able to help them fill out housing application forms. Another patient who was dealing with food insecurity shared how their PN was “able to send [her] details and locations for food pantries.” Other patients shared experiences of times in which their PN went above and beyond what they expected. “One time I was having a really bad flare up and didn’t have anyone to watch my son while I visited the doctor. [My PN] volunteered to take care of him for a few hours so I could see the doctor.”

##### 3.2.1.4. Theme 4. Strong relationships and empathy.

Patients repeatedly mentioned the relationships they were grateful to forge with their PNs. Many PNs were able to help simply provide support to patients in times of need. As one patient summarized, “She (my PN) gave me hope. She always listens to me. She gives me advice. When she speaks with me she makes me feel happy.” Another patient shared, “.the program has been great. [My PN] is very nice and she calls to check in on me which I really appreciate to know someone is looking out for me.” Yet another patient made a comment that she feels like she can relate to her PN “.like a daughter. She gives me a lot of hope when I’m feeling down. She encourages me to take care of myself. She always calls to check in on me and makes me feel better when I’m feeling lonely.”

#### 3.2.2. Areas for improvement

The overwhelming response from patients was positive. However, when prompted for constructive feedback, eight patients provided examples of ways their navigator was not able to meet their needs. Seven of these responses made reference to inadequate communication from their PN wishing the student had contacted them more regularly to check-in or remind them of appointments. Two patients said they had wanted help with filling out housing forms that their PN was not able to assist with, however recognized that this may be out of the scope of their role. Similarly, one patient felt she was unaware of how the navigator could be of assistance. Finally, one patient wished their navigator was able to accompany her to appointments more regularly and felt that her PN was not “competent enough in terms of medical knowledge.”

## 4. Discussion and conclusion

### 4.1. Discussion

This cross-sectional study focused on the patient experience of a medical student patient navigation program in Brooklyn, New York. While prior studies have demonstrated the efficacy of patient navigation programs in health outcomes on a number of different metrics, few have explored the patient perspective on these programs. Furthermore, the use of medical students as PNs has not been widely implemented despite calls from academic organizations to expand their use [30]. Our findings demonstrate high levels of patient satisfaction with employing medical students as PNs for patients with significant barriers to care. Students were able to be most helpful to patients in scheduling appointments, contacting patients’ doctors, filling prescriptions, answering medical questions, addressing life stressors, and providing emotional assistance. As a result of the program, many patients expressed feeling more cared for, feeling more motivated to care for their health, and feeling better understood by their healthcare team. The importance of these results is bolstered by the strong association between a number of these psychosocial factors and clinical outcomes (including medication adherence) [31–33].

Some have focused on the benefits of student navigation programs on medical education by providing students with longitudinal, health systems-based, value-added patient experience [34–37]. However, we argue that the use of medical students as navigators confers a number of unique benefits to patients as well. First, by implementing a PN program using a number of medical students, the case-load per navigator can be diffused. Second, medical students are eager to gain early access to patient experience. Thus, compared to other types of navigators, students may be more proactive in reacting to patient needs. Additionally, clinics wishing to implement a PN program need not hire an additional staff member who requires training, salary, and clearance. As students already exist within the university medical system, they have a basic understanding of medical jargon and systems and may require less training than other potential PNs. While these factors may not hold true everywhere, compared to other recipients of our PN grant, our students were able to begin seeing patients months before any hired navigators at the four other sites. Furthermore, students’ basic medical knowledge allows them to serve a more expansive role than non-clinical navigators [38]. It should be noted that no head-to-head data exist comparing student navigators to others. Nonetheless, in conjunction with other similar models that have improved health outcomes with the use of student navigators [31], our patient-centered data indicate high levels of satisfaction from patients with this model.

### 4.2. Strengths and limitations

The present study is not without limitations. First, given that this was a pilot program, there was variation in quality and duration of follow up based on patient and PN factors. We did not seek to quantify the amount of time spent on each patient, instead choosing to analyze the program as a whole. Some patients had weekly contact with their navigators, while others only interacted with them a number of times whether due to lack of need or lack of follow up by either party. This was reported as feedback from a number of patients as noted above. Furthermore, as the present program enlisted medical students of varying backgrounds and interests, naturally, some pairings developed more therapeutic relationships than others and we did not conduct individual evaluations by navigator. Second, while our response rate was acceptable at over 63%, our results may be confounded by nonresponse bias. Next, it is important to note that this study focused solely on the patient’s experience of the program rather than looking at direct disease outcome measures. Numerous studies have demonstrated that PNs can be quite efficacious. We, therefore, chose to examine patient perspectives specifically. It should also be noted that patient experience responses tend to be positively skewed and should be used as a starting point for further improvements in patient care [39,40]. Finally, this study was limited in scope to a single outpatient setting in a minority urban population. The patients served have many health problems and significantly lack health resources. By design, this may contribute to selection bias as our program chose to intervene for our most at-risk patients rather than a random sampling. These are patients that have a clear need for a program like this, while other patient populations may not demonstrate the same robust results. At the same time, PN programs have been shown to be most beneficial in low income populations, especially in communities that have been historically disadvantaged [16]. Our study corroborates this data and serves to strengthen the support for employing PNs for underserved patients.

### 4.3. Conclusion

In this study of 44 patients enrolled in a novel medical student patient navigation program, patients expressed high levels of satisfaction across a number of different domains. Patients’ specific needs were met in the vast majority of cases spanning appointment scheduling to emotional support. As a result, most patients reported feeling more cared for by their healthcare team, felt their healthcare team better understood their challenges, and were more motivated to better care for their health. Our study builds on existing data that demonstrates that PN programs can be mutually beneficial for students and patients. While additional longitudinal data is needed to better assess follow up and disease-specific outcomes, our initial data suggest that patients, at least subjectively, benefit greatly from the use of students as care coordinators. With this model’s strong educational benefit, further research is warranted to better assess the efficacy of students compared to lay navigators in achieving patient-centered disease outcomes.

### 4.4. Practice implications

Patients from lower SES backgrounds continue to struggle to navigate America’s increasingly complex healthcare system. PNs have been proven to be an effective, low cost, and personal way to address this growing issue. Medical student patient navigation is an easily scalable approach that can be used in medical schools throughout the country in a variety of departments. Medical schools throughout the country have been moving towards earlier clinical experience for their students. Additionally, medical students often lack longitudinal care opportunities early in their training. Many medical students have the opportunity to work with at-risk patients in a student-run free clinic (as is the case in our institution). However, these encounters are limited to a single visit and lack the critical longitudinal follow up that allows for a more robust understanding of the issues these patients face in accessing care [41]. Choosing students as navigators not only provides tremendous educational benefit, but fills a crucial gap in care for underserved patients. In addition to the stated roles outlined above, students may have the opportunity to identify contextual errors (errors that result from overlooking patient contextual factors) [42] and learn to avoid these errors in their future practice. This program’s preliminary success has led to its expansion into other departments in our hospital and is being considered for inclusion into the medical student curriculum at our institution.

Future directions for this model are vast and promising. These may include: (1) providing medical students with additional training and clarifying communication expectations; (2) a social worker to whom students can refer patients for more complex issues; (3) reflection exercises for students to process their patient experiences and share resources; (4) tailored matching of student-patient pairs based on both patient and student characteristics and/or interests; (5) patient-led teaching sessions about barriers they face to care.

Our hope is that with further expansion and research, medical student-based patient navigation can become a pillar of both medical student education and patient care.

## Figures and Tables

**Table 1:** Demographic Representation of Patients.

Demographics	Number of Patients (N = 44)
**Sex**	
*Male*	7
*Female*	37
**Ethnicity**	
*White*	2
*Black or African American*	41
*Asian*	1
**Primary Language**	
*English*	41
*Spanish*	1
*Creole*	1
*Chinese*	1
**Insurance**	
*Medicare/Medicaid*	25
*HealthFirst*	4
*MetroPlus*	4
Other*	11
	**Age Values (years old)**
**Age**	
*Range*	21 – 91
*Median*	42
*Mean*	45

**Table 2:** Survey.

1. On a scale of 1 to 5 please rate your satisfaction with the PN program (1 = very dissatisfied, 2 = dissatisfied, 3 = neutral, 4 = satisfied, and 5 = very satisfied)
2. On a scale of 1 to 5 how do you feel about the following statements (1 = strongly disagree, 2 = disagree, 3 = neutral, 4 = agree, and 5 = strongly agree):
a. As a result of my experience in the PN program I felt more cared for by my healthcare team.
b. As a result of my experience in the PN program I felt more motivated to better care for my health
c. As a result of my experience in the PN program I felt my healthcare team better understood the challenges I face in daily life
3. I am most interested in receiving help from a PN with (check all that apply*):
4. My PN was able to help me with (check all that apply*):
a. Getting in touch with my doctor
b. Filling prescriptions
c. Scheduling appointments
d. Answering medical questions
e. Helping arrange transportation to clinic visits
f. Reminding me of appointments
g. Filling out forms
h. Providing emotional support
i. Providing additional clarification from clinic visits
j. Other
*Answer choices a-j were presented for both question 3 and 4
5. Free response:
a. What was the most helpful aspect of having a PN?
b. Do you have any stories that stood out to you from your navigator experience that you would like to share?
c. Were there any additional areas of support that you felt were not met by your PN?

## Appendix A

**Table A.1:** Individual patient responses to survey questions 3 and 4.

	Getting in touch with my doctor	Filling prescriptions	Scheduling appointments	Answering medical questions	Helping arrange transportation to clinic visits	Reminding me of appointments	Filling out forms	Providing emotional support	Providing additional clarification from clinic visits
Patient 1		✓		✓					✓
Patient 2									
Patient 3	✓	✓	✓	✓		✓		✓	✓
Patient 4	✓	✓	✓						✓
Patient 5		✓				✓			✓
Patient 6	✓	✓	✓	✓		✓			✓
Patient 7	✓	✓	✓	✓	✓	✓		✓	✓
Patient 8	✓	✓	✓	✓		✓			✓
Patient 9			✓						✓
Patient 10		✓	✓			✓			
Patient 11	✓	✓	✓	✓		✓		✓	✓
Patient 12	✓	✓	✓						
Patient 13	✓	✓	✓	✓	✓	✓	✓	✓	✓
Patient 14									
Patient 15									
Patient 16	✓								
Patient 17	✓		✓			✓			✓
Patient 18									
Patient 19	✓		✓	✓		✓	✓	✓	✓
Patient 20	✓	✓		✓		✓	✓		✓
Patient 21									
Patient 22	✓			✓				✓	✓
Patient 23			✓						
Patient 24	✓	✓	✓	✓	✓	✓	✓	✓	✓
Patient 25			✓	✓		✓			
Patient 26	✓	✓	✓	✓	✓	✓	✓	✓	✓
Patient 27						✓			
Patient 28	✓	✓	✓		✓	✓			✓
Patient 29	✓	✓	✓	✓		✓			✓
Patient 30	✓	✓	✓	✓		✓	✓	✓	✓
Patient 31		✓	✓						
Patient 32									
Patient 33	✓	✓	✓						
Patient 34									
Patient 35	✓	✓	✓	✓		✓			✓
Patient 36	✓	✓	✓	✓		✓	✓	✓	✓
Patient 37	✓		✓	✓		✓	✓	✓	✓
Patient 38	✓	✓	✓	✓		✓	✓	✓	✓
Patient 39	✓			✓					✓
Patient 40	✓	✓	✓	✓	✓	✓	✓		✓
Patient 41	✓	✓	✓	✓			✓	✓	✓
Patient 42	✓	✓	✓	✓		✓		✓	✓
Patient 43	✓		✓	✓					
Patient 44	✓	✓	✓	✓		✓		✓	✓

* A gray background indicates the patient was interested in receiving help while the background is white if the patient was not interested in receiving help in this area. A check mark indicates the PN was able to help with this area.
